# The use of self-expanding metallic stents in the management of benign colonic obstruction: a systematic review and meta-analysis

**DOI:** 10.1007/s10151-024-02959-7

**Published:** 2024-07-19

**Authors:** Armin Fardanesh, Jayan George, Daniel Hughes, Stavroula Stavropoulou-Tatla, Pawan Mathur

**Affiliations:** 1 Department of General Surgery, Royal London Hospital, Bart’s Health NHS Foundation Trust, London, England, UK; 2 Department of General Surgery, Doncaster and Bassetlaw Teaching Hospitals NHS Foundation Trust, Sheffield, England, UK; 3 Division of Clinical Medicine, University of Sheffield, Sheffield, England, UK; 4 Department of UGI Surgery, Royal Berkshire NHS Foundation Trust, Reading, England, UK; 5 Department of Plastic Surgery, St George’s University Hospitals NHS Foundation Trust, London, England, UK; 6 Department of General Surgery, Royal Free London NHS Foundation Trust, Royal Free Hospital, London, England, UK

**Keywords:** Benign, Large bowel obstruction, Benign colonic obstruction, Stenting, Self-expanding metal stents, Meta-analysis

## Abstract

**Introduction:**

Patients presenting with large bowel obstruction (LBO) frequently undergo emergency surgery that is associated with significant morbidity. In malignant LBO, endoscopic approaches with placement is a self-expanding metal stent (SEMS), have been proposed to prevent emergency surgery and act as a bridge to an elective procedure—with the intention of avoiding a stoma and reducing morbidity. This systematic review aims to assess the quality and outcomes of data available on the use of SEMS in benign causes of colonic obstruction.

**Methods:**

This systematic review was performed using the Preferred Reporting Items for Systematic Reviews and Meta-Analyses (PRISMA) guidelines, and the protocol was registered on Prospero (ID: CRD42021239363). PUBMED, MEDLINE, HMIC, CINAHL, AMED, EMBASE, APA and Cochrane databases were searched. Studies were assessed for quality utilising the MINORS criteria. Pooled odds ratios with 95% confidence intervals (95% CI) were calculated using random effects models.

**Results:**

Sixteen studies were included for analysis. 300 patients were included with an average age of 68, and a male predominance of 57%. The quality of the papers included were at risk of bias. The pooled rate of technical success of procedure was 94.4% (95% CI 90.5–96.8%) The pooled rate of clinical success was 77.6% (95% CI: 66.6–85.7%). Adverse effects were low, with perforation 8.8% (4.5–16.6%), recurrence 26.5% (17.2–38.5%) and stent migration 22.5% (14.1–33.8%).

**Discussion:**

This systematic review demonstrated that SEMS for benign colonic obstruction can be a safe and successful procedure. The utilisation of SEMS in malignant disease as a bridge to surgery has been well documented. Whilst the limitations of the data interpreted are appreciated, we postulate that SEMS could be utilised to decompress patients acutely and allow pre-operative optimisation, leading to a more elective surgery with less subsequent morbidity.

## Introduction

Acute colonic obstruction is a surgical emergency most often caused by colorectal carcinoma, with up to a quarter of patients with colorectal malignancy presenting with acute large bowel obstruction (LBO). Whilst, over 60% of cases can be attributed to malignancy, acute colonic obstruction can also be secondary to benign diseases such as diverticulosis and inflammatory bowel disease, as well as a late postsurgical complication [1–3]. Emergency surgery typically involves a laparotomy and formation of a stoma, either an end colostomy or diverting stoma, and is associated with mortality rates of up to 30% and high risks of future anastomotic leak [4]. The rates of stoma reversal range from 19.2–69% [5–8].

Self-expanding metallic stents (SEMS) have been utilised since 1991 as a minimally invasive alternative to emergency surgery in acute LBO [9, 10]. The use of SEMS has been established as a “bridge to surgery” in patients with malignant disease; allowing decompression of the obstruction and avoiding an emergent surgery with high mortality rates [11]. The vast majority of stomas are never reversed [12]. Establishing luminal patency, allows for patient optimisation for surgery, with pre-operative planning; and facilitates the possibility of a single operation with resection and primary anastomosis, eliminating the need for a stoma in up to 90% [2, 13, 14]. The second recognised indication for SEMS is as a palliative treatment intervention in patients unlikely to be fit for a major operation or with advanced metastatic disease [15].

A more topical use of SEMS has been in obstruction secondary to benign causes of LBO such as diverticular disease or inflammatory bowel disease. Thus far, there has been varying success and uptake of SEMS in benign diseases owing to the reported high risk of complications such as stent migration and perforation [16].

This systematic review aims to investigate the efficacy of SEMS for all benign colorectal obstruction and to determine its safety and feasibility as a reliable possible “bridge to surgery” intervention.

## Methods

This systematic review protocol was registered on Prospero (ID: CRD42021239363). A comprehensive literature search was performed from PUBMED, MEDLINE, HMIC, CINAHL, AMED, EMBASE, Cochrane databases and APA in June 2023. Searching was structured and centred around PICO [17]. Population: (adult, obstruction, benign), Intervention: (stent, types of stent), Comparisons or control group: (not applicable), Outcomes of interest (complications, safety, success, readmission, mortality, migration, stoma, perforation, reoperation, refractory, bridge to surgery). We also hand-searched original papers in gastrointestinal journals. Study selection was performed by two authors (JG and AF) and conflicts were decided by a third author (DH). Recommendations from the preferred reported items of systematic reviews and meta-analysis (PRISMA) were followed in our systematic review [18]. The searches included all results up until June 2023.

### Eligibility, selection, data extraction and quality assessment

We included all retrospective and prospective papers, or abstracts which reported on outcomes of benign colorectal stenting where full data was present. Only adult human studies published in English or where translation was possible were included.

Exclusion criteria—(1) studies including a paediatric population (2) studies that only included malignant causes of colonic obstruction (3) articles with less than 10 benign cases (4) abstracts without full case information (5) animal studies.

Extracted data from each study included study information including: study design, the country of origin, the year of publication, demographic data of the cohort, type of stent placed, procedure and stent (position of stent, stent length, number of stents, stent diameter, interventional radiology or endoscopic approach, surgeon/radiologist involvement). In studies with a mix of malignant and benign cases, these were interrogated to extract benign case outcomes only and included in the meta-analysis if they had over 10 benign cases.

Outcomes extracted included the following:

*Primary outcomes* Technical success (defined as successful deployment of SEMS) and Clinical success (defined as the subsequent successful decompression of LBO).

*Secondary outcomes* Post-procedure outcomes (mean stent indwelling time, follow-up, bridge to surgery, time interval and nature of surgery, stoma avoided, outcome at 30 days and long-term success at one year) and complications (perforation, recurrence, migration, death, cause of death, adverse events, number of complications > grade 3 Clavien-Dindo).

Quality assessment was performed by two authors (AF and SS) using the MINORS criteria [19], with a third author utilised to overcome any disagreements.

### Statistical analysis

All statistical analysis was performed using R Foundation Statistical software (R 3.6.3). A formal meta-analysis of proportions was performed using a random effects model incorporating the DerSimonian-Laird method. Results were visualised through Forest plots. *I*2 value was calculated to assess the degree of heterogeneity amongst the included studies. Heterogeneity was determined as low (< 25%), middle (25–75%) or high (> 75%) as per the *I*2 value. Results were deemed of statistical significance if *p* < 0.05.

## Results

**Fig. 1:**
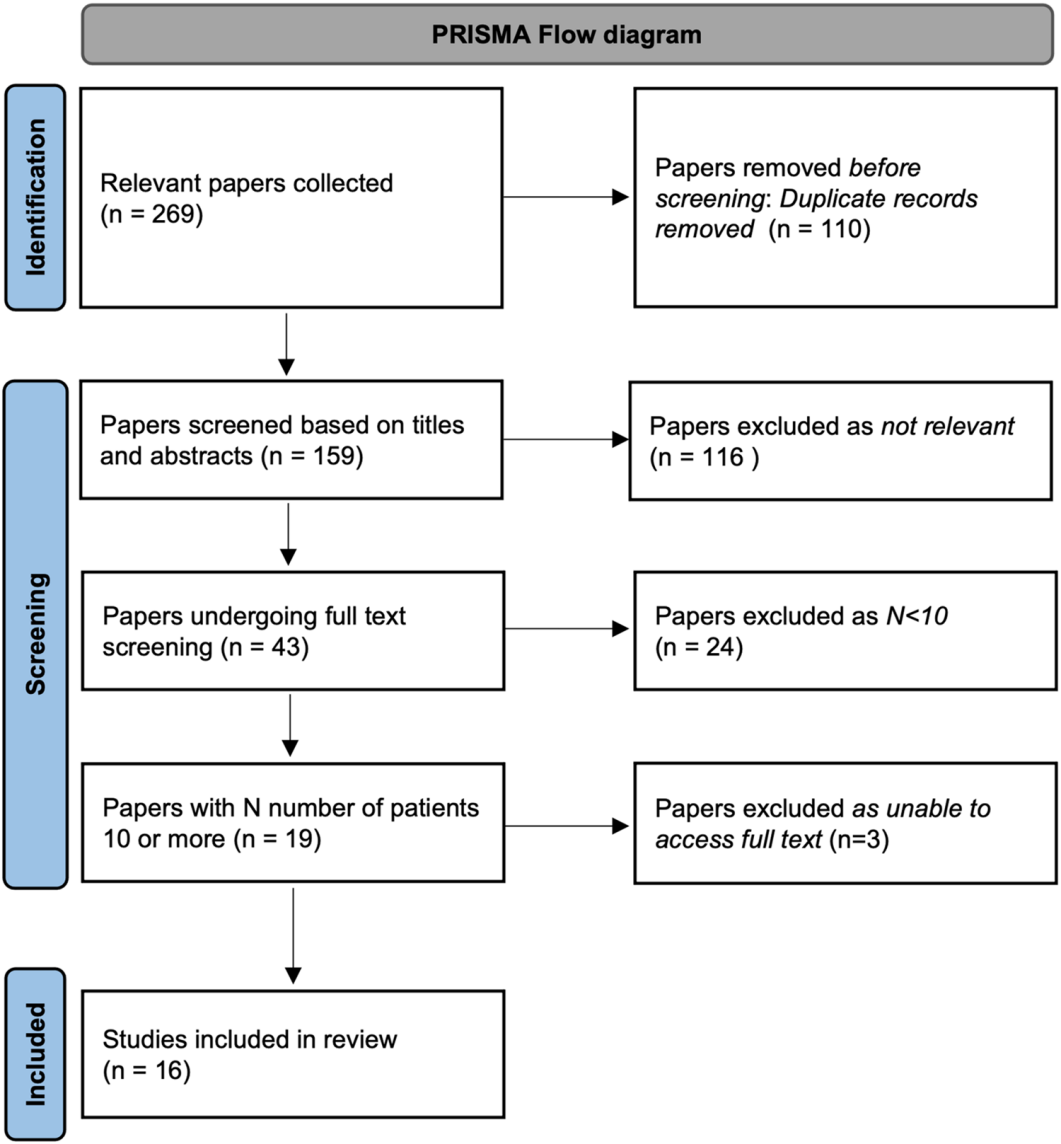
PRISMA flow diagram demonstrating the systematic process from paper collection to studies included in the review

The initial search identified 269 papers, 110 duplicates were removed. Abstract screening of 159 identified 43 papers for full-text screening. 24 were excluded, 3 were unable to be sourced. 16 studies were included in the systematic review. PRISMA diagram is shown in Fig. 1.

Dual reviewer screening was used, with a third reviewer as a decider. The background characteristics are summarised in Table 1.

**Table 1:** Demographics: each paper breakdown of patient demographics

Author	Year Published	Country of Origin	Total number of patients	Mean age of patients (range)	Number of male patients	Number of female patients
Boyle et al.	2015	UK	17	Not recorded	Not recorded	Not recorded
Caruso Angelo et al.	2015	Italy	16	76 (64–78)	10	6
Cereatti et al.	2016	France	29	60 (19–82)	19	10
Dai et al.	2010	Germany	14	62 (44–73)	10	4
Demarquay et al.	2008	France	19	87 (73–93)	8	11
Forshaw et al.	2005	UK	11	65 (46–89)	7	4
Hong et al.	2020	Korea	12	61 (43–89)	12	0
Keranen et al.	2010	Finland	21	69 (34–89)	9	12
Kohler et al.	2014	Austria	36	Not recorded	Not recorded	Not recorded
Lamazza et al.	2013	Italy	10	73 (52–83)	7	3
Mackay et al.	2011	Scotland	15	Not recorded	Not recorded	Not recorded
Park et al.	2015	Korea	14	62 (31–84)	5	9
Paul et al.	2002	Spain	10	63 (51–74)	6	4
Small et al.	2007	USA	23	66 (41–97)	9	14
Vanbiervliet et al.	2012	France	43	68 (58–78)	24	19
Yan et al.	2021	China	10	Not recorded	Not recorded	Not recorded
Total: 16			300	68 (19–97)	126 (56.8%)	96 (43.2%)

Where documented “not recorded” the paper did not specify patient demographics

The total number of patients in the review was 300. Sex and age were able to be extracted in 222 patients (56.8% male and 43.2% female) and mean age 68 (range: 19–97). Aetiology was documented in 300 patients. The most common aetiology was an anastomotic stricture 136/300 (45.3%) followed by IBD 63/300 (21.0%) (Tables 2, 3).

**Table 2:**
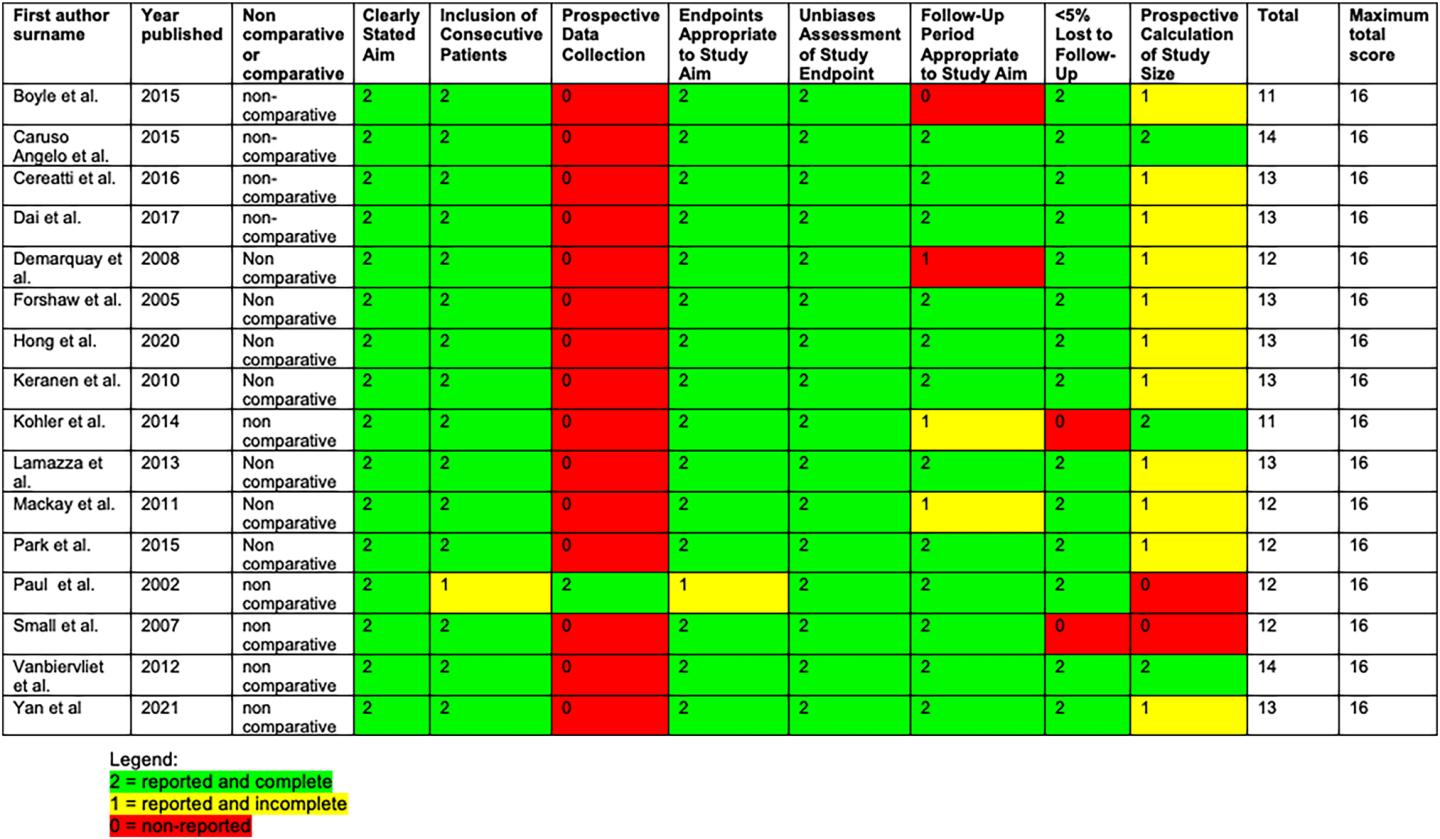
Quality assessment: each paper quality assessment as per the MINORS criteria

**Table 3:** Aetiology: representing the breakdown of each benign cause of LBO

First author surname	Year published	Country of origin	Total number of patients	Diverticular	Anastomotic	IBD	Crohn’s	Ulcerative colitis	Other colitis	Extrinsic	Ischaemic	Post radiation	Other
Boyle et al.	2015	UK	17	13	0	0	0	0	0	4	0	0	0
Caruso Angelo et al.	2015	Italy	16	0	16	0	0	0	0	0	0	0	0
Cereatti et al.	2016	France	29	0	29	0	0	0	0	0	0	0	0
Dai et al.	2010	Germany	14	0	13	0	0	0	0	0	0	0	1
Demarquay et al.	2008	France	19	6	6	0	0	0	0	0	5	2	0
Forshaw et al.	2005	UK	11	4	4	0	0	0	1	0	1	0	0
Hong et al.	2020	Korea	12	0	12	0	0	0	0	0	0	0	0
Keranen et al.	2010	Finland	21	10	8	0	2	0	0	0	0	1	0
Kohler et al.	2014	Austria	36	0	14	20	0	0	0	0	0	2	0
Lamazza et al.	2013	Italy	10	0	10	0	0	0	0	0	0	0	0
Mackay et al.	2011	Scotland	15	6	N/A*	N/A*	N/A *	N/A *	N/A *	N/A*	1	N/A *	0
Park et al.	2015	Korea	14	0	10	0	0	0	0	0	1	3	0
Paul et al.	2002	Spain	10	2	4	0	0	0	4	0	0	0	0
Small et al.	2007	USA	23	14	3	2	1	0	0	0	0	3	0
Vanbiervliet et al.	2012	France	43	0	0	40	0	0	0	0	2	1	0
Yan et al.	2021	China	10	0	7	1	0	0	0	0	0	0	2
**Total**			300	55	136	63	3	0	5	4	10	12	3
**%**				18.3%	45.3%	21.0%	1.0%	0.0%	1.7%	1.3%	3.3%	4.0%	1.0%

N/A * = unable to extract exact breakdown of aetiology. Data not included in totals

### Quality of studies and risk of bias

**Fig. 2:**
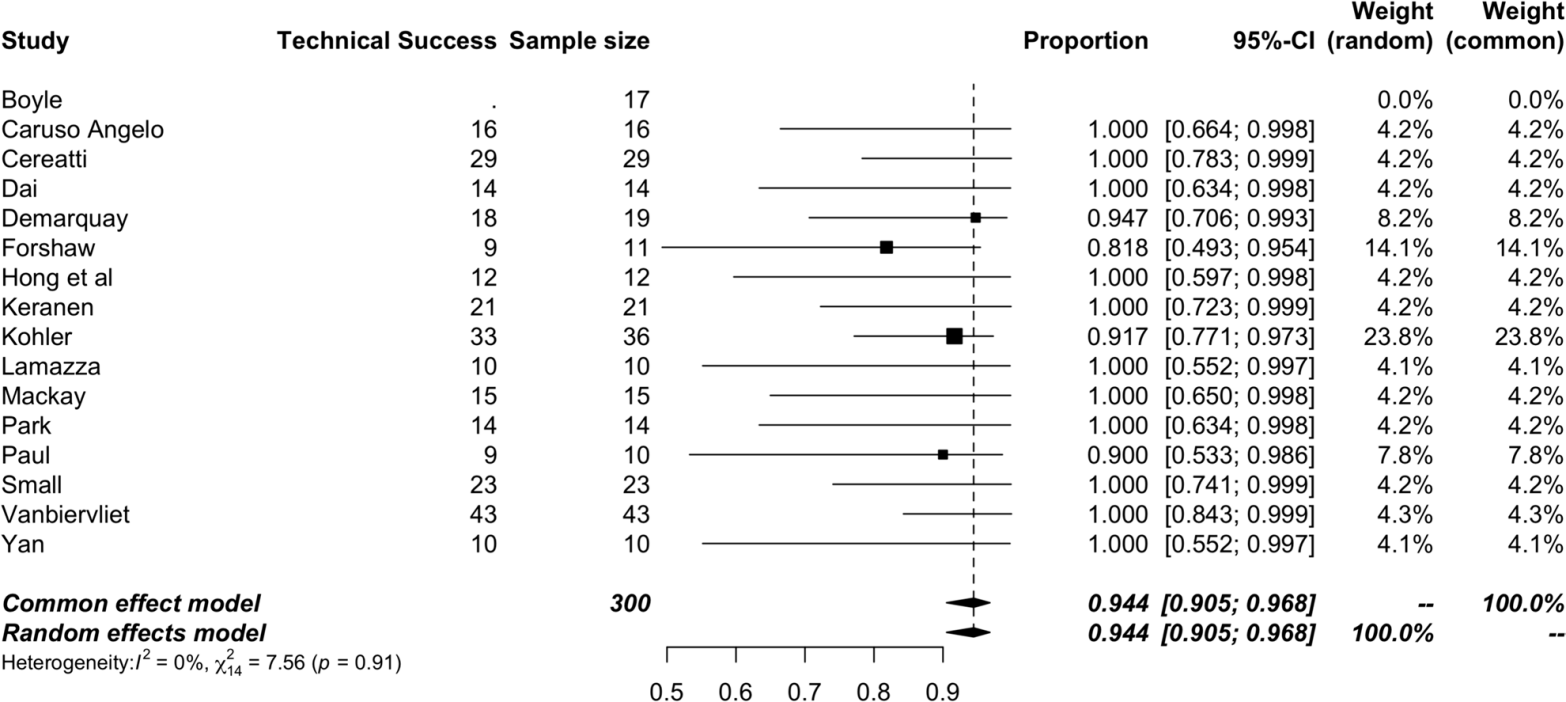
Forrest plot of technical success

The 16 studies included were all non-randomised and non-comparative studies. The majority (15/16) were retrospective. All papers were subjected to significant bias and scored less than 16. Quality assessment in full in Table 3 (Fig. 2).

### Technical success

Technical success defined as the successful deployment of SEMS, reported on was in 16 studies. The pooled rate was 94.4% (95% confidence intervals 90.5–96.8) with a low degree of heterogeneity (*I*^2^ = 7.6, *p* = 0.91).

### Clinical success

**Fig. 3:**
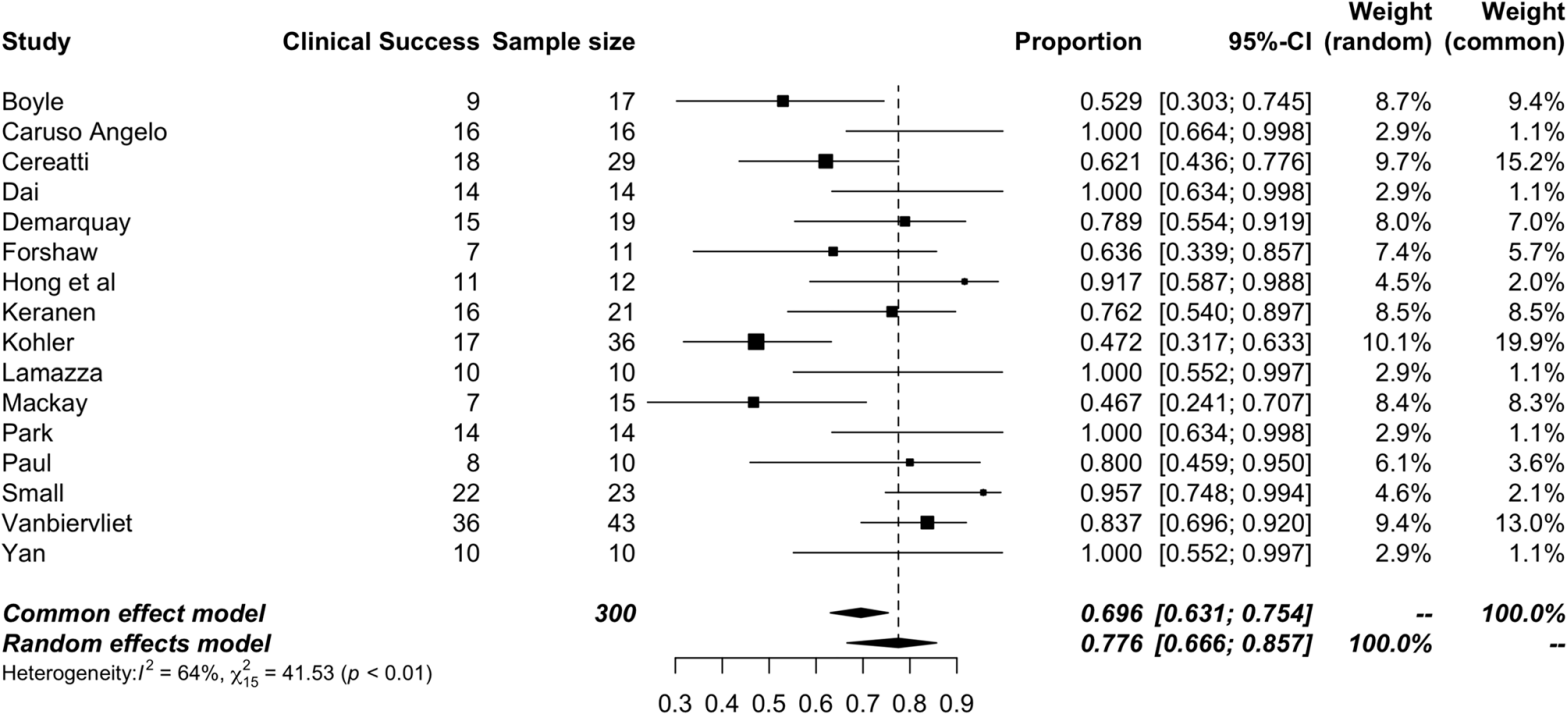
Forrest plot of clinical success

Clinical success defined as successful decompression of LBO was reported on in 16 studies. The pooled rate was 77.6% (CI: 66.6–85.7%) with a middle degree of heterogeneity (*I*^2^ = 41.53, *p* < 0.01) (Fig. 3).

### Bridge to surgery

Data reported for the bridge to surgery was widely variable, with a high degree of heterogeneity. Data extraction for statistical analysis was unable to be performed. Of 16 studies, four studies did not record postprocedural outcomes at all. The 30-day outcome was only clearly documented in one study. “Bridge to surgery” was only mentioned in a quarter of studies. Unfortunately, data regarding the nature of all surgery and whether a stoma was avoided was inconsistently reported and unable to be extracted.

### Adverse events

#### Perforation

There were 16 studies that included perforation which contributed to 300 cases. The pooled rate was 8.8% (4.5–16.6%), with an *I*^2^ of 35.5 (*p* < 0.01).

#### Recurrence of large bowel obstruction

There were 14 studies that included recurrence which contributed to 273 cases. The pooled rate was 26.5% (17.2–38.5%) with an *I*^2^ of 36.77 (*p* < 0.01).

#### Stent migration

There were 14 studies that included stent migration which contributed to 273 cases. The pooled rate was 22.5% (14.1–33.8%) with an *I*^2^ of 40.12 (*p* < 0.01).

#### Death

There were 14 studies that reported in depth on further adverse outcomes. Of 273 cases, there were only 2 deaths. Both of which in patients who suffered from post-procedural perforations and were reported to have refused surgery.

## Discussion

Emergency surgery in patients with LBO is associated with higher morbidity, mortality, as well as the likelihood of a permanent stoma [4, 5]. Stenting in malignant LBO may be beneficial, however, international guidance for left-sided resectable obstruction does not have a clear consensus of the benefits as more high-quality published data is required [20]. SEMS can potentially convert an emergency surgery into a planned elective procedure. SEMS is well-established in malignant colorectal disease and has been shown to have advantages as a bridge to surgery over emergency surgery [21].

Our study is the largest and most recent study to summarise the use of SEMS in benign colorectal conditions. This meta-analysis demonstrates the technical success in these patients is high with 94.4% which is similar to previously published data [22]. Clinical success in our study is also high at 78% and is comparable to recently published malignant palliative large bowel obstruction data of 97.1% [23]. Serious adverse events such as perforation were low. These results were similarly seen in a large case series for large bowel obstruction (including malignant and benign) [24].

Stenting for colorectal cancer as a bridge to surgery has been shown to be safe in achieving 90% of obstructions with clinical success over 70% [25]. The CReST randomised clinical trial for colorectal stenting for obstructing left-sided colorectal cancer demonstrated that stenting as a bridge to surgery reduces stoma formation [26]. This paper shows that benign colonic stenting achieves a similarly high clinical and technical success rate. There are still questions over the use of SEMS that remain. SEMS can decompress high-grade colonic obstruction however this is likely to be only a temporising measure as opposed to long-term options and only in certain patient groups [25, 27]. There is a high degree of heterogeneity and there is not a uniform format of reporting in the majority of the studies included in the synthesis.

Malignant colonic stenting is established in routine practice of obstructed patients and there is a large body of evidence to support its use in a certain subgroup of patients. Malignant colonic stenting is used as a bridge to surgery and results in lower operative time, lower overall complication rates (33.9–37.8% versus 51.2–54.8%) as well as lower rates of temporary (28.8–33.9% versus 46–51.4%) and permanent (22.2% versus 35.3%) stoma [25, 28–30]. It is not clear as to whether there would be similar results with benign colonic stenting. There was not enough high-quality data to extract from the papers included in this analysis.

Whilst the plausible benefits of stenting with SEMS have been discussed, many centres opt for endoscopic dilatation as the first-line means of conservative intervention for relieving acute LBO, due to the procedural simplicity and low risk profile. Studies have demonstrated similar clinical success rates when compared with SEMS [31]. Nevertheless, dilatation is suited predominately to shorter, anastomotic strictures [32], with narrow stenosis. Dilatation yields poorer in the presence of active inflammation—seen often in diverticular disease—or fistulous disease [32]. Furthermore, endoscopic dilatation frequently requires multiple repeated procedures, and thus is not preferred when the goal of the intervention is to serve as a single bridge to definitive surgery. In addition, refractoriness to dilatation has been described in approximately a fifth of cases [33]. However, studies have demonstrated that cases that have struggled with endoscopic dilatation, are not predictive of poor outcomes with SEMS [34]. Thus, proposing that failure after dilatation can be managed with SEMS, rather than operative management—which is particularly important for patients too frail for surgery.

One of the most common complications of the SEMS insertion is stent migration, seen in up to 27% of cases in this systematic review. Poor long-term patency has been frequently discussed as one of the major downsides of SEMS as a definitive treatment option for LBO due to benign lesions [31]. However, the high technical and clinical success of the procedure with effective decompression of obstruction suggests stent migration may be a late phenomenon, due to sufficient dilatation of the bowel lumen at the site of the causative lesion, and is occasionally not even noticed by patients, [35]. In the setting of utilising SEMS as a means to bridge to an early elective resection of the causative lesion as well as indwelling stent—late stent migration is less of a concern.

Bridge to surgery has already been suggested by the ESGE (European Society of Gastrointestinal Endoscopy Clinical Guideline) for colonic malignancy [36]. The guidelines suggest utilising a bridge to elective surgery in patients over the age of 70 and are ASA grade III and above. Furthermore, the planned timeframe for surgery is 5–10 days after stent insertion. Whilst this is taken into the context of malignancy, a 5–10 day interval allows the patient to be optimised before surgery, but also is short enough to reduce the risk of stent-related complications such as fibrosis and migration. Other centres have utilised a bridge of median of 17 days in malignant cases, allowing for a higher proportion of definitive laparoscopic resection, with reduced morbidity to patients [37].

The evidence suggests that the risk of migration in malignant cases is lower, hence allowing for a more generous length of bridge, we suggest utilising a similar approach in benign cases. A short bridge will allow preoperative optimisation for patients, including for management of medical co-morbidities, pre-operative colonic cleansing, further radiological assessments and treatment of possible infection [36, 37]. We anticipate that effective preoperative planning and optimisation along with a timed-elective procedure will reduce the post-operative morbidity, and reduce the need for a stoma, with higher rates of primary anastomoses following resection.

Selection of suitable patients is prudent, as the other benefit of utilising SEMS, involves providing safer treatment options for patients who are unlikely to tolerate drastic colorectal surgery even in the elective setting—such as the elderly and co-morbid. Thus, stenting can be used in a more palliative approach, to provide longer-term relief of LBO. This is even more relevant in benign causes of LBO, where there is less risk of spread or advancement of disease as opposed to malignancy. With satisfactory decompression, these patients may then be offered further endoscopic procedures, or stent changes but will ultimately avoid a general anaesthetic and drastic operation.

It must be noted that in order to offer endoscopic interventions, there is a pre-requisite of adequately trained endoscopists or interventional radiologists. In a third of the papers interrogated in this study, the procedure was performed by an interventional radiologist. The required equipment and the presence of suitable SEMS in the department are also limiting factors that may lead to surgery being favoured.

Our review has highlighted the lack of consistent reporting in the literature to be able to extract data on bridge to surgery. It would be of great use nationally and internationally to have a database which included all the relevant information outlined in this review as well as clearer definitions of bridge to surgery and follow-up to give more understanding to the efficacy of SEMS in benign colorectal conditions.

### Limitations

A major challenge with this review is the variability in reporting. Whilst technical success was reliably described, studies frequently did not specify exact outcomes for specific patients and there was a paucity of data on longer-term outcomes. As mentioned in the results section, there was very limited reporting on “bridge to surgery” and fewer studies reported surgical outcomes after stenting, Furthermore, we were unable to conclude outcomes at 30 days or one year.

Moreover, for our data to be sufficiently accurate, we did not include studies that had less than 10 cases and thus would not have enough power to be statistically significant. This may have led to some smaller studies with compelling results being missed, however, this would have been unlikely to affect our meta-analysis results. Furthermore, few studies described the outcomes of both malignant and benign cases together and it was not possible to distinguish the outcomes of the benign cases alone, therefore these were not included in the analysis.

## Conclusion

The use of SEMS in benign LBO carries high technical and clinical success rates with low complication rates. Studies thus far are heterogeneous and it is not possible to draw sufficient conclusions from them. We propose the utilisation of a wide-scale database to analyse current practice and to help in drawing conclusions.

## Data Availability

Further data can be provided at request from the corresponding authors.
